# Science museum educators’ views on object-based learning: The perceived importance of authenticity and touch

**DOI:** 10.1177/09636625231202617

**Published:** 2023-11-02

**Authors:** Tirsa de Kluis, Sanne Romp, Anne M. Land-Zandstra

**Affiliations:** Leiden University, The Netherlands

**Keywords:** authenticity, object-based learning, replica, touch

## Abstract

Museum educators play an important role in mediating visitors’ museum experiences. We investigated the perspectives of science museum educators on the role of touching authentic objects and replicas in visitors’ learning experiences during educational activities. We used a mixed-methods approach including surveys with 49 museum educators and interviews with 12 museum educators from several countries in Europe. Our findings indicate the importance of context when presenting museum visitors with objects. Participating museum educators based their choices for including authentic objects or replicas in educational activities more often on narrative and context than on the authenticity status of an object. In addition, educators used various definitions of authenticity, which may hinder the discussion about the topic within the field.

## 1. Introduction

Science museums are one type of venue where visitors can engage with science, through visiting exhibitions, interacting with interactive exhibits, meeting scientists, and contributing to scientific research (National Research Council, 2009). The unique value of science museums is often their authentic collection of objects such as fossils, taxidermied animals, (historical) scientific instruments, and technological inventions. It is generally assumed that authenticity and the opportunity for visitors to touch the objects have a positive effect on visitors’ museum experiences (Dima et al., 2014; Wilson et al., 2017). However, evidence for how this affects visitors’ learning experiences is sometimes lacking or inconclusive (Hampp and Schwan, 2014a, 2014b; Minogue and Jones, 2006). Furthermore, the learning process in science museums during educational activities is often mediated by museum educators. Their views and the choices they make regarding the use of objects have an impact on the learning experience of visitors. In order to better understand the attitudes and opinions of science museum educators regarding authenticity and touch during educational activities in science museums, we surveyed and interviewed museum educators about these topics.

### Object-based learning in science museums

Many people remember the time that museums were a place to look at objects while keeping your hands on your back. However, the focus of science museums has changed significantly over the past years, mimicking other trends in the field of science communication from one-way communication toward more interactive forms of science engagement (Friedman, 2010). The field of informal science education has since established itself as an environment for learning science outside of the school system, overlapping and complementing formal science education (National Research Council, 2009). With regard to museum collections, it was recognized that visitors would not acquire the same knowledge from just looking at objects as curators or researchers would (Evans et al., 2002). Subsequently, the focus of museum learning and design shifted from letting the object speak for itself to the relationship the visitor has with the object (Evans et al., 2002). This relationship can be established by interacting with the object, for example, by incorporating object-based learning. This interaction with objects is often supported by educational activities such as guided tours, family activities, science shows, or show-and-tell, where visitors are specifically encouraged to actively engage with objects. The strength of object-based learning is that it evokes multiple senses and stimulates interactive or even inquiry-based learning (Paris and Hapgood, 2002). The active interaction with museum objects can inform, motivate, and inspire visitors, facilitating learning opportunities (Chatterjee et al., 2015).

In considering learning in science museums, the focus is usually broader than just knowledge acquisition. Several frameworks have been developed specifically for learning in informal settings such as museums, with a lot of overlap (e.g., Allen et al., 2008; National Research Council, 2009). These frameworks include learning outcomes such as interest, creativity, curiosity, acquiring skills, identifying with science, and attitudes toward science. These broad learning outcomes are relevant for science museums and other informal learning settings since, in those places, the focus is often more on sparking curiosity and excitement than on conveying facts and concepts (Shouse et al., 2010).

### Authenticity

Authentic objects are often at the core of the learning opportunities offered in science museums. They can play a role in the visitors’ learning experiences by triggering curiosity or providing concrete examples (Chatterjee et al., 2015; Tran, 2008). The authenticity of museum objects can be defined in different ways. For example, as described by Evans et al. (2002), the authenticity of an artifact can mean that the object is an original and not a copy. In contrast, a natural object can be considered authentic when it originates from nature. Other criteria for authenticity are historical significance, uniqueness, rarity, and charisma (Hampp and Schwan, 2014a; Van Gerven et al., 2018). In this study, we adopt a broad meaning of the term authenticity: an object can be considered authentic if (1) it is a real object originating from nature; (2) the object has a long history; (3) the object is unique; or (4) the object has belonged to a famous or important person. In contrast, replicas can be described as copies of an original object. They can either be (almost) indistinguishable such as direct casts or exact reconstructions or less realistic such as replicas of scientific instruments that are made to show the workings of an apparatus.

Although evidence suggests that visitors appreciate authentic objects, it is unclear if and how authentic objects have a different impact on visitors’ learning experiences than replicas. In some studies, authentic objects have an impact by linking the museum experience to abstract concepts (Chatterjee et al., 2015) or by fostering curiosity (Bunce, 2016). In the latter study, Bunce (2016) found that participants asked more questions about an object when they judged it to be authentic. However, other researchers found that, in some situations, visitors do not experience a difference between interacting with authentic objects or replicas (Hampp and Schwan, 2014a; Latour and Lowe, 2011; Penrose, 2018). Hampp and Schwan (2014a) showed that visitors’ perception of the relevance of an object was not influenced when it was disclosed to be either a replica or an authentic object. The feelings an object evoked were more dependent on the type of object. Similarly, Latour and Lowe (2011) argue that a well-constructed replica of an artwork may have a similar impact on visitors as the original. According to Penrose (2018), the context and story surrounding the object are more important than the authenticity itself.

### Touching objects

Although historically, museum visitors were not allowed to touch the objects, we have seen a shift toward more tactile interaction with objects. In formal education, touching objects during classes seems to increase students’ understanding and engagement (Smith, 2016a). Educational activities in museums also often incorporate the possibility of touching museum objects to facilitate interaction and learning (Pye, 2007; Wilson et al., 2017). When studying the importance of touching objects for creating interest, Dohn (2010) showed that hands-on learning with museum objects is a key factor in gaining immediate interest. Living specimens of fish, shrimp, and crabs offered students highly personal hands-on experiences. These experiences generated interest since students experienced it as fun, fascinating, and exciting but also creepy to handle live fish. In addition, Novak et al. (2020) showed that museum visitors who were allowed to touch and handle objects showed higher levels of autonomy and were better at recollecting the information.

### Museum educators

When investigating learning experiences in science museums, it is important to realize that these learning experiences are often facilitated by museum educators: staff members who develop and deliver the museum’s educational activities (Tran, 2008). The use of objects is an intrinsic part of the work of science museum educators to help visitors appreciate and understand science (Tran, 2008). Therefore, museum educators’ perspectives, opinions, and practices shape the learning experience of visitors, just like they do for learning in formal school environments (Kyriakides et al., 2013). Although we know a bit about how visitors experience authenticity and handling objects, as described in the previous paragraphs, studies on the perspective of museum educators on the importance of object authenticity and handling objects during educational activities are limited. Therefore, the aim of this research is to answer the following research question: What is the perspective of science museum educators on the role of authentic objects and replicas on visitors’ learning experiences during educational activities? This research question was split into three sub-questions:

1. How do museum educators define authenticity?
2. What is the perspective of museum educators on the role of authentic objects and replicas within educational activities?
3. What is the perspective of museum educators on touching objects and its influence on learning during educational activities?

## 2. Materials and methods

### Mixed-methods research

In this exploratory study, we used a mixed-methods approach to explore science museum educators’ perceptions and opinions on the use and touch of authentic objects and replicas in science museums’ educational activities. Both qualitative and quantitative data were collected in parallel, using surveys and interviews. In this way, connections between these two data types could strengthen the conclusions (Shorten and Smith, 2017).

### Participants

Forty-nine museum educators responded to the survey, and 12 participated in an interview. Participants for the survey and the interviews were recruited using convenience sampling within the Netherlands and other countries in Europe (making use of the network of the authors). Social media was used to distribute the survey as well. In addition, participants of the survey were asked to participate in the interviews and vice versa. Because of the anonymous nature of the survey and to preserve privacy, we do not have information about the overlap between interviewees and survey participants. However, because of the complementary nature of the survey (more general questions) and the interview (more in-depth discussions), this overlap should not have impacted our results. Unfortunately, six participants did not fully complete the survey, resulting in some missing data, in particular regarding their demographics. Although the number of participants in the survey is small, we believe that the exploratory nature of this study justifies the sample size. For the interviews, we reached saturation of findings after the 12 interviews.

Participants were museum educators working at science museums in Europe that had authentic objects available to use in educational activities. Forty-three (out of 49) of the survey participants, and all 12 interviewees, were involved in performing and developing educational activities. The most highlighted topics in the museums where participants are employed are related to nature or natural history (38 survey participants and 8 interviewees) and Physics/Chemistry/Mathematics/Science (23 survey participants and 7 interviewees).

To describe the sample, demographics were collected. For the survey, 24 museum educators were employed in the Netherlands, 19 outside the Netherlands such as in the United Kingdom (3 educators) and Finland (3 educators), and of 7 educators, this was unknown (Table 1). Because of the location of the authors in the Netherlands, we were able to recruit more Dutch museum educators than from other countries in this convenience sample. A majority of participants were female (67%). At the time of the survey, 41 participants were active as museum educators or developers, 2 were not, and of 6, it is unknown. The mean years of working experience was 12.9 years (*SD* 8.4). They were mostly involved with teaching children aged 8–10 years old (36 educators) and 11–13 years old (34 educators).

**Table 1.** Survey participant characteristics.

Characteristics (no. of respondents)	Mean (*SD*)
Experience in years (*N* = 41)	12.9 (8.4)
Gender (*N* = 43)	Frequency
Male	12
Female	29
Other	2
Employed in (*N* = 43)	
Belgium	2
Denmark	2
Finland	3
Poland	2
The Netherlands	24
United Kingdom	3
Other	7
Museum topics (*N* = 43)^a^	
Physics/Chemistry/Mathematics/Science	23
Ethnology	5
Biology	29
Geography	16
(Natural) History	9
Other	10
Involved age groups (*N* = 43)^a^	
4–7 years old	20
8–10 years old	36
11–13 years old	34
14–16 years old	20
>16 years old	14

SD: standard deviation.

Six educators did not finish the survey. *N* is the number of respondents.

a Multiple answer options are possible.

Of the 12 museum educators that were interviewed, 5 worked in a museum in the Netherlands, and 7 outside the Netherlands. The mean years of working experience was 8.5 years (range 0.5–22 years). Next to being a museum educator, four interviewees had experience with science communication, five used to be school teachers, and six studied a science-related topic.

Informed consent was acquired before the survey and interviews; survey answers were anonymous; interview data were processed confidentially, no names were saved in the final database.

### Data collection

The online survey contained 28 questions, combining multiple-choice, Likert-type scale, and open questions (Supplemental Material 1). Questions were designed based on literature research and expert consultation. We pilot-tested the survey with museum educators. The survey was distributed in both Dutch and English. At the beginning of the survey, we provided the participants with a description of authentic objects (such as fossils, objects of famous people, or antique instruments) and replicas (representationally realistic replicas such as exact casts and less realistic ones), to make sure all participants answered the questions with the same concept in mind. First, the perceived importance of using objects during educational activities was examined, by asking what type of objects educators used and in what way. Second and third, the educators’ opinions were asked about the opportunity to touch objects during educational activities and its possible effects on visitors, and about object authenticity using five 5-point Likert-type-scale questions each. Fourth, the positive or negative effects of replicas compared with authentic objects were researched by giving two scenarios, where educators had to choose whether they would use an authentic object or a replica and why, followed by open questions about the added value and barriers of both. All Likert-type-scale questions were followed by an option to explain their answers.

The interviews were conducted through video calls. They started with a general introduction, during which the museum educators discussed their background and experience. Then, their thoughts about the definition of authenticity were discussed, by discussing the predefined definition of authenticity. The importance of objects, authenticity, and touch were discussed too, by asking for typical examples of how objects were used and if objects are important in educational activities. Finally, museum educators were asked about the application of objects during educational activities. They described situations in which they would use authentic objects or replicas. A complete overview of the interview scheme is presented in Supplemental Material 2.

### Analysis

#### Surveys

Multiple-choice and Likert-type-scale questions were analyzed through descriptive statistics. A qualitative analysis was performed on the open questions of the survey. Inductive coding was used for the answers to these questions. Answers were labeled with subcodes that were then combined into categories. Table 2 provides an overview of the codebook, and the complete codebook can be found in Supplemental Material 3. Another researcher coded 10 surveys (20%) independently. The intercoder reliability was 67%. Disagreements mainly related to unspecific terms in the codebook or too much interpretation of the statements. Unspecific codes were changed and answers were re-coded, to reach consensus.

**Table 2.** Overview of survey codebook. The extended version is available in Supplemental Material 3.

Category	Codes	Description	Example
Goal	Engagement Learning (Museum) Experience Museum’s right to exist	The goal of using objects during lessons or activities	“It can prompt interest and curiosity” (S010)
Use	Authentic objects Replicas Similar objects Educational collection No specification object	What kind of objects and in what way educators use objects during lessons or activities	“If this is an object from the official museum collection I would only let people look at it, as close as possible and then in a small display case” (S027)
Consideration touch or show	Show authentic Touch replica	If the educator would rather allow visitors to touch a replica or to only look at an authentic object	“The idea that such a tooth was in a real mouth and that it allowed millions of years ago a dinosaur to eat leads to a nice conversation” (S030)
Touchable authentic objects	Object characteristics Impact visitor Museum characteristics Other	The added value and barrier of touchable authentic objects	“The feeling you get in touch with something special or unique” (S002)
Touchable replicas	Object characteristics Impact visitor Museum characteristics Other	The added value and barrier of touchable replicas	“With a replica you can observe details of the object better” (S021)

#### Interviews

After transcription, the interviews were coded both deductively and inductively: some codes were predefined based on the theoretical concepts of authenticity discussed in the introduction, while other codes emerged from the data. Five percent (45 segments) of the total sample was coded separately by another researcher. Half of these segments were selected randomly, and half were selected because of doubts about the initial coding. An agreement of 44% was found, and after discussion, consensus was reached about the subsample. To correct for the disagreement from lack of context, a complete interview was coded separately by this second researcher. An agreement of 60% was found. Table 3 contains an overview of the codebook. The complete codebook can be found in Supplemental Material 4. In this article, survey and interview quotes in Dutch were translated into English.

**Table 3.** Overview of interview codebook. The extended version is available in Supplemental Material 4.

Category	Code	Description	Example
Institute	Role	Role the museum educators take on during the museum lesson or activity	“So it is not a monologue, it always has to be a dialogue between the participants and the guide.” (I7)
	Object choice	Process of choosing objects for educational activity	“Well we will start by looking at what we need in order to teach the kids what we want to teach them. So if we are going to teach them the one where they count the teeth, then of course we will have some of the different animals with the different amounts of teeth in their mouth.” (I5)
	Barriers	Barriers to the use of objects that stem from the institute	“Of course, I can think that I want to show a diplodocus tail, but that is not possible since that is way too costly and we don’t have that.” (I3)
Object	Authenticity	How the educator defines the term authenticity; Reasons for choosing either an authentic object or a replica; Way in which educators distinguish authentic objects and replicas for their audience	“I think normally we would only call it authentic if it is really something from nature and not something man-made.” (I5)
	Use	Impact of touching/showing an authentic object/replica	“I think it is just ‘wow, I touched something that was made 2000 years ago by someone’. I think that that makes a difference.” (I13)
	Goal	Goal educator has in mind when visitors touch/are shown an authentic object/replica	“You can connect it very well to normal life because the dinosaur only has teeth in the front and not in the back. So you can ask how did it chew and what would happen if you had the same teeth and eat a carrot, or something like that.” (I17)
	Barriers	Barriers to the use of objects that stem from the object itself	“Because it is usually rare or fragile we can’t give them the real stuff.” (I7)
Visitor	Learning	Way in which learning occurs because of the use of objects	“Especially when you have a replica that can be held. Then you use even more senses, that works much better than having a real T. rex tooth in a display.” (I8)
	Barriers	Barriers to the use of objects that stem from the visitor	“With teenagers, they usually are hesitant to hold things . . . they consider rocks to be dirty.” (I3)

## 3. Results

### Authenticity

#### Definition of authenticity

In the surveys, a description with examples of authentic objects and replicas was given at the start of the survey to make sure all participating museum educators answered the questions with this in mind. In the interviews, the definition of authenticity, being (1) a real object originating from nature; (2) the object has a long history; (3) the object is unique; or (4) the object has belonged to a famous or important person, was discussed more in depth. It was found that all participating museum educators worked with a slightly or even completely different interpretation of the term authenticity. Most educators responded that they recognized at least parts of the proposed definition, particularly the criterion originating from nature, which was unanimously seen as a valid reason to consider an object authentic. One educator stated: “I think normally we would only call it authentic if it is really from nature and not something man-made” (I1). In contrast, 75% of the interviewed educators initially had doubts about considering an object authentic because it belonged to a famous person. After discussing this, it often appeared that they do see it as authentic, but they did not have these kinds of objects in their museums. In addition, some mentioned that when a replica has belonged to a famous person, this can actually transform into an authentic object as well: “If it [a replica] has been in the possession of Darwin, is it still an authentic object? Yes, it seems to be authentic but it actually is a replica. So then it is actually both” (I10).

A little over half of the interviewees (58%) explicitly mentioned that when an object has a long history, this would be a valid reason for it to be authentic. Interestingly, this long history was also mentioned by some to be a way in which replicas can become authentic: “We also have replicas in the collection which are very special objects because they are really old replicas. In that case they become authentic, but in a different way” (I3). Considering an object authentic because it is unique or rare was mentioned less often. This criterion seemed to influence the degree of the object’s authenticity but was not a make or break point when trying to define an object’s authenticity. This was discussed by four educators, of which one mentioned:

The term rarity also plays a role here I think [. . .] Also, if it has a long history and many things have happened to it, then there are very few objects that encountered all those events. So I understand that it becomes more authentic then. (I2)

Interestingly, two (17%) of the interviewed educators considered exact replicas to be authentic objects too, having differing explanations for this. One mentioned that it was more practical to use one term for both the authentic objects and exact replicas that were used during the educational activity: “We had a discussion and authentic ended up being a much simpler way to say that we have both real specimens and exact copies, but it is not just any copy” (I7). The other explained that exact replicas can still create an authentic experience because they look identical to the objects which would be considered authentic based on the definition used in this study.

#### Distinction between authentic objects and replicas

Surveyed educators stated that it is important that visitors are aware of the authenticity of an object, and that in the ideal case, a replica is handled next to a displayed authentic object. Almost all (92%) interviewees mentioned that it is important to make a clear distinction between authentic objects and replicas, to prevent misinforming visitors about the objects or decreasing their engagement. One interviewee explained,

For all dinosaur skeletons that we have had for a long time it is hardly distinguishable what is real and what is not. [. . .] Whereas, at the [. . .] last dinosaur we placed, we deliberately chose to show the difference between the 3D-printed bones and the real bones. [. . .] you want to prevent people from thinking “oh you always find a complete skeleton of a dinosaur,” because that is not the case. (I2)

In contrast, 42% of the interviewed educators nuanced their answers by discussing situations in which it is not fitting to distinguish between authentic objects and replicas on the basis of similar reasons. One interviewee explained: “I think that it can sometimes be slightly distracting if the conversation then becomes about the replica versus real conversation rather than the learning outcome that you might be hoping for” (I6). In addition, these educators mentioned that it often does not matter to the visitor if the object is authentic or a replica as long as it fits well into the story that is told.

However, all educators did agree that they would never lie about the authenticity of an object if they were directly asked about it. In the cases that they were replicas, educators also often saw this as a way to discuss objects on another level, by explaining why they were showing a replica instead of an authentic object or by discussing the process of making the replica.

#### Using authentic objects or replicas

Of the surveyed educators, 57% used both authentic objects and replicas during their activities. Typical examples of objects they used were fossils, teeth, and animal fur. Replicas could be displayed next to an authentic object and were used for more fragile items. Fourteen percent of the surveyed educators only used replicas that are as realistic as possible, such as an exact cast or copy. When discussing the use of objects during educational activities more in-depth, the interviewed educators indicated that they most often showed authentic objects or gave the opportunity to handle replicas. Which ones they used was heavily dependent on the activity aim and the prior knowledge and interest of the target audience.

**Figure 1.**
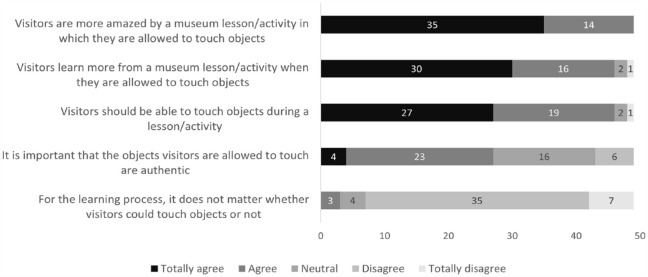
Opinions of museum educators concerning the use of authentic objects during educational activities, compared with realistic replicas (*N* = 49).

Figure 1 shows the opinions on object authenticity of the museum educators who participated in the survey. The statements in the survey compared authentic objects with highly realistic replicas, such as an exact cast. Most educators (totally) agreed on the importance of looking at authentic objects even without touching them and that visitors become more enthusiastic when authentic objects are used. However, opinions differed about whether authentic objects have a different impact on learning compared with replicas: 31% of the participants (totally) agreed that visitors learn more when authentic objects are used, and 27% (totally) disagreed.

In the interviews, 83% of the museum educators mentioned that an authentic object can have an added value over a replica. Some educators did not have clear arguments for this but just explained it as being a bonus. Fifty percent of the educators further emphasized that authentic objects can increase the amazement of visitors:

If you say “yes, that is a real tusk of a mammoth,” then they really say “wow, really? It is so heavy! Did it really carry this on its head?” Well, if you would give them a replica then it would not make such an impression. (I10)

Another important characteristic of the use of authentic objects is the connection with history that can be made when observing or interacting with them: “Sometimes, an authentic object can bring across better the realization of how old an object actually is, while a replica gives more insight in how something functions or how something was made” (I5).

In the interviews, museum educators often mentioned the added value of the authenticity of an object, but stated that authenticity on its own is not enough. Eighty-three percent of the educators explained that the narrative around the objects plays an important role in how the objects are perceived by visitors. For example, one of them said: “I think [the story] is the key. If you tell enthusiastically and passionately about a plastic menalite, then that still really does a lot” (I3). Because the narrative influences the way visitors perceive objects, this also heavily affects how the objects are presented to them.

#### Authenticity and touch

Surveyed museum educators did not all agree whether they would rather have visitors touch a replica than have them only look at an authentic object. The survey showed that using touchable authentic objects during educational activities brings both potential added value and barriers. Impact on the visitor, for instance, on the learning experience, was mentioned as one added value (78%). Other added values were objects’ characteristics, being unique or a link with history (35%), as well as museum characteristics (e.g., appreciation of the collection, 10%). The vulnerability of touchable authentic objects (82%) and the related accessibility within the museum (49%) were seen as barriers. For example, 22% of the surveyed educators mentioned as a barrier the fact that visitors are unable to tell or understand what the authentic object is. In the interviews, it was found that replicas were mostly appreciated for their educational value. The appearance of the replicas played a very important role since this can be made to be so perfect that it adds to their educational value and can sometimes even give them an added value over authentic objects.

When surveyed museum educators were asked about the added value and barriers of touching realistic replicas, the impact on the learning experience was mentioned often (61%). Moreover, replicas are less vulnerable and less expensive and a museum can possess more of them (59%). The majority of possible barriers were related to object characteristics, being unauthentic and fragile (43%). Other barriers were loss of interest and disappointment of visitors (35%) and museum-related barriers, such as the price of replicas and the risk of theft (20%). Sixteen percent of the surveyed educators experienced no barriers to using touchable replicas. In addition, in the interviews it was discussed that replicas can be of educational value when they are used together with the authentic object:

We have Magdeburg hemispheres in our collection. [. . .] we have the real ones, we can show those in the museum, but the children will work with replicas. They do not look very real [. . .] but [visitors] can still investigate how they work. (I9)

In the survey, a similar question was included. Museum educators were given a hypothetical situation and were asked if they would rather display a genuine Tyrannosaurus tooth in a showcase during an educational activity, or let visitors touch and pass around an exact replica of the tooth. Opinions again diverged: 31% of the educators chose displaying a real tooth while 55% chose handling the replica.

#### Objects and learning experience

##### The role of objects in establishing engagement

All surveyed educators (*n* = 49) agreed with the statement that it is important to use objects in general during an educational activity. Forty-five percent of the educators’ justifications for this statement included that objects can lead to engagement by triggering interest, emotion, grabbing attention, and surprising the visitors. In addition, another 45% of the justifications included that objects enhance the learning process because they inform people. Furthermore, objects can create a successful museum experience by making it more meaningful and memorable and making the past tangible (55%). Finally, educators mentioned that objects are the museum’s right to exist (8%).

During the interviews, the learning experience in relation to objects was discussed more in-depth. Interestingly, the actual learning of new knowledge or facts was often deemed unimportant by the participating museum educators. They were more interested in giving the visitor a positive experience with science and therefore were more focused on engagement, interest, and attitudes. One of the educators described it as:

I always find it more interesting to see what it does with the attitude of the children, or what kind of emotions it evokes, and I do not find it interesting at all if at the end of the lesson they have remembered exactly what a meander is or what obsidian is. (I3)

All interviewed museum educators agreed that objects play an important role in achieving this engagement of the visitors. The main reason for this is that objects amaze and interest visitors. One way in which objects trigger engagement is by enabling a connection between the life of the visitor and the object, according to 42% of the interviewed educators. One interviewee stated,

I find the [human] skulls very interesting and nice to use, because they are close to the students. It is recognizable. It is about us, about humans, so by definition about them. (I2)

##### The effect of touch on the learning experience

Most of the surveyed museum educators (86%) allowed visitors to touch and hold objects. When asked to explain further, 59% of the surveyed educators mentioned that visitors were allowed to touch the authentic objects, but only under supervision. In addition, 53% of the surveyed educators mentioned that the presence of replicas allowed visitors to touch and examine objects themselves. Furthermore, 63% of the surveyed educators showed objects to visitors, and 55% allowed visitors to look at or touch an object without supervision. A few educators mentioned that it depends heavily on the activity.

**Figure 2.**
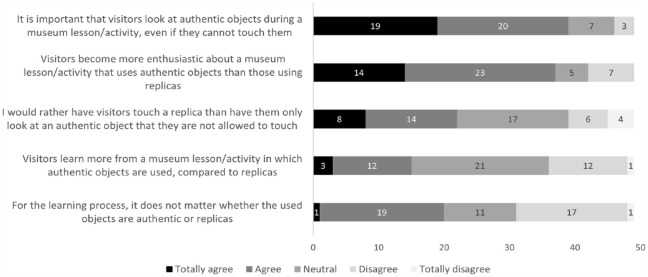
Opinions of museum educators concerning the ability to touch objects during educational activities (*N* = 49).

When asked about their opinions about touching objects, the majority of surveyed museum educators (totally) agreed that visitors are more amazed or excited by an educational activity in which objects can be touched, compared with an activity in which visitors can only look at objects. Moreover, they agreed that visitors learn more and that touching objects is a prerequisite for any museum activity (Figure 2). Educators in this study were less unanimous about the need for these touchable objects to be authentic.

In the interviews, all participating museum educators agreed on the importance of touch during their educational activities. Yet, they gave different reasons for the impact on learning. Seventy-five percent of the interviewed educators mentioned that touching or handling objects can increase the learning experience because visitors can explore the objects using multiple senses:

When I talk about dinosaur bones that are now fossilized and I show them a fossilized tree [. . .] they can touch the fossilized tree and they understand it is not wood anymore. Now it is stone, but it still has the shape of wood. So then everything about dinosaurs gets clearer. (I12)

Also, touching and handling the object can create a more memorable experience or accommodate visitors with different learning preferences or physical, or sensory disabilities. In addition, some educators mentioned that it increases visitors’ scientific literacy because they can mimic the work of scientists when handling the objects themselves. Scientific literacy in general was mentioned by 42% of the educators to be an important goal for using objects.

## 4. Discussion

In science museums, authentic objects are utilized to contribute to visitors’ broad learning outcomes such as a better understanding of science, interest in science, or curiosity. This learning process is mediated by museum educators. In this study, we investigated the perceptions and opinions of museum educators regarding the use of authentic objects in educational activities in science museums through a survey and interviews. In particular, we explored how educators define authenticity within the science museum context, how they perceive the differences between authentic objects and replicas, and what their perspectives are regarding allowing visitors to touch objects and the impact that has on the learning experience.

### Definition

During the interviews, not all museum educators meant the same thing by the term authenticity. Most of them offered a narrow definition by only considering an object authentic if it originated from nature instead of the broader range of definitions found in literature including unique, original objects, or man-made objects of historical significance. This could be caused by the fact that most of the interviewees were affiliated with museums where objects originating from nature are most prevalent. Most of the museum educators in our study could also agree with one or more other criteria for authenticity such as a long history, uniqueness, or the object belonging to a famous person but mentioned that they used those criteria less. The fact that the term authenticity does not have one overarching definition is not surprising. Previous research defined authentic objects as multivoiced entities, which means that there are multiple ways in which the objects can be considered authentic (Evans et al., 2002).

Interestingly, some participants also considered exact replicas to be authentic objects, depending on the appearance of the replicas and the techniques used to make them. In addition, objects that were once considered replicas could slowly acquire a “status” of authenticity, for example, due to being in the collection for a long time. The notion that an object can acquire a certain essence that results in visitors and educators considering it as (more) authentic was also seen in previous research by Rossi (2010) and Van Gerven et al. (2019). In these studies, replicas could be perceived as authentic objects because they had a specific background story or belonged to a national celebrity. In this way certain replicas can acquire authenticity by their history, longevity, or because they belonged to a specific person.

### Authentic objects and replicas

#### Distinction

A little over half of the surveyed educators indicated that they consider the authenticity of a museum object important for educational activities. Moreover, the majority of educators mentioned in the survey that it is important to show authentic objects during educational activities to inspire visitors, even if they cannot touch them. This was endorsed during the interviews, where almost all participants found it important to distinguish between authentic objects and replicas. However, during half of the interviews, participants also discussed situations in which this was not beneficial for the learning experience or engagement of the visitors. It seems according to the interviewees, that the importance of distinguishing between authentic objects and replicas is dependent on the narrative around that object. From studies among visitors, it appears that visitors do not always distinguish between authentic objects and replicas, or at least appreciate them equally. For example, Hampp and Schwan (2014b) found that visitors of a science exhibition did not appreciate replicas less than authentic objects. This appreciation was, however, influenced by the context in which the object was presented.

#### Authentic objects

The benefits that educators mentioned in the survey of using authentic objects over replicas were that authentic objects are unique and contain history. Previous studies showed that object history is indeed appreciated by visitors, especially children (Van Gerven et al., 2018). Furthermore, according to the educators in our study, it makes visitors appreciate the museum and its collection, which was seen in other studies as well (Hampp and Schwan, 2014a; Soren, 2009). Moreover, many educators stated that touching an authentic object makes visitors more enthusiastic about the museum lesson or activity than when replicas are used. This corresponds to previous research, showing that authentic objects increase the engagement and interest of visitors (Bunce, 2016). Authentic objects can be seen as more valuable than replicas, and visitors are more willing to touch those objects (Frazier et al., 2009). In addition, some participants referred to the museum’s “right to exist” as a reason to prefer authentic objects. They state that museums have an obligation to show authentic objects as much as possible because that is why they exist in the first place (Hampp and Schwan, 2014a). Some barriers to using authentic objects, according to museum educators, are that these objects are often vulnerable and that sometimes museum rules prohibit the use of authentic objects in educational activities.

#### Replicas

With regard to the use of replicas, respondents mentioned that being able to engage with an object is more important than authenticity, which was in line with earlier research (Di Franco et al., 2015). Also, according to educators in our study, replicas can even have a more beneficial effect on the learning experience and on long-term impact such as remembering the lesson. Di Franco et al. (2015) found that students experienced touchable 3D printed replicas as more engaging than real archeological artifacts. However, in their study, there were characteristics of the artifacts that were easier understood from the real artifact, such as weight, while other characteristics were easier understood from the 3D replica, such as texture. Wilson et al. (2017) showed that museum visitors indicated that their visit was more fun and educational when they were allowed to touch replicas. In addition, educators in our study mentioned that replicas are less vulnerable than authentic objects and they can be handled and reproduced more easily so that multiple visitors can use them at the same time.

### The impact of touching objects on the learning experience

#### Impact of using authentic objects and replicas in educational activities

Many of the stated reasons for using and touching objects in educational activities were related to the perceived impact on the learning experience of visitors. We define the learning experience broadly to include not only learning new knowledge but also developing curiosity, interest, and attitudes (Allen et al., 2008; National Research Council, 2009). Almost all participating educators agreed that using objects has an impact on learning because it helps visitors understand what things look like or how they function. But also more indirectly, using objects may prompt curiosity or interest, grab attention, trigger amazement, or start a conversation. This perception is also supported by research among visitors themselves, where the use of objects triggered visitors to start asking questions (Bunce, 2016). As curiosity is associated with better learning outcomes (Kang et al., 2008; Loewenstein, 1994), the use of objects may increase learning as well. In addition, a strong emotion (both positive and negative) can increase the learning experience (Tyng et al., 2017). Interestingly, most of the interviewed museum educators were less interested in how much new knowledge visitors gained through the use of objects but wanted to make the experience memorable by focusing on attitudes, curiosity, and emotions, giving visitors a positive experience with science. This aligns with the broad framework of learning science in informal settings (National Research Council, 2009).

When discussing the impact on the learning experience of authentic objects versus replicas, the participating educators mentioned different aspects of these objects. Authentic objects seem to be better suited to ignite a sense of wonder, excitement, and curiosity, while replicas that can be handled are capable of helping to understand how things work or function. Many educators think that a combination of the two would be perfect. This also connects to the fact that the selection of real objects and replicas for educational activities is often dependent on the context and the learning goals of the activity. Museum educators found it important to create a connection with the personal lives of the visitors. This finding resonates with the study of Penrose (2018), who emphasized that personal connection with an object is important when conveying the story behind that object. He states that, when visitors identify with an object, this makes them more receptive to the story the museum tries to convey.

#### Impact of touching objects on the learning experience

In a previous section, we discussed how replicas are sometimes preferred over authentic objects because they allow visitors to touch and handle objects. According to the participating educators, touching and handling objects has a positive impact on the learning experience. This fits within the framework of haptic exploration and embodied cognition where cognitive learning is considered to be highly impacted by bodily experiences (Novak et al., 2020). Novak et al. (2020) saw something similar when surveying visitors who were allowed to touch objects versus visitors who could only look at the objects or pictures of the objects. Many educators even agreed that educational activities that allow visitors to touch objects are more attractive to visitors than activities where they can only look at objects. Some educators mentioned the use of multiple senses as a driver for learning. Several other studies and authors have supported the claim that learning through multiple senses is beneficial (Chatterjee, 2008; Gallace and Spence, 2008; Shams and Seitz, 2008).

Another related argument was the observation that hands-on learning, in this case actively engaging with objects, is beneficial. This is consistent with previous studies, indicating that hands-on experiences with objects create interest and engagement, which is important for the learning experience (Dohn, 2010; Renninger et al., 1992; Smith, 2016b). Several interviewees touched upon this argument as well, stating that touching and handling objects can allow visitors to mimic the work of scientists and increase their scientific literacy. In a study with high school students, Achiam et al. (2016) found that students could be encouraged to think and argue like paleontologists when offered a skeleton of a modern bird and an exact cast of an archaeopteryx fossil. In addition, Land-Zandstra et al. (2020) also showed how presenting families with an authentic object and a question could trigger them to start reasoning about the object. These examples and the comments from the interviewees show how authentic objects in science museums can play a role in increasing this aspect of scientific literacy.

### Limitations and future research

Some limitations of this study should be considered. First, by distributing the survey via the personal contacts of the researchers, a convenience sample was created. However, by distributing the survey via social media as well, this bias was minimized as much as possible. In addition, a nonresponse bias could have occurred. Educators who participated in the survey and interview are likely those who like to express their opinions. Because of this, the opinions of less outspoken educators could have been overlooked. We tried to overcome this by approaching many educators using a personal email and following up regularly.

Second, because of the exploratory nature of this research, the sample sizes were quite small and there was a bias for natural history museums in the Netherlands. A larger sample size could potentially generate stronger conclusions and more generalizable findings, especially regarding the importance of authenticity since the opinions related to this topic were very diverse. In addition, most participants in our study worked predominantly with young age groups. This does align with the fact that for many science museums (school) children and families are their main target group.

Future research should try to reach out to a larger representative sample to determine how well our findings match the opinion of a larger group of science museum educators. Moreover, science museums other than natural history museums, and museums outside the Netherlands, should be investigated. In addition, it would be interesting to compare our findings for science museum educators with the opinions of educators in different types of museums, such as culture and history museums.

## 5. Conclusion

By using a mixed-methods approach in this study, it was possible to both give a general overview of the perceptions of museum educators on the use of authentic objects as well as investigate their reasoning behind these perceptions. This complements previous research by both enforcing earlier findings and presenting new arguments brought forward by the participating museum educators.

One of the main findings of this study was the profound importance of narrative and context. Museum educators base their choice for objects during educational activities more on this than on whether an object is authentic or a replica. This implies that research into this topic might be too heavily focused on authenticity and instead should concentrate more on the way in which objects are presented. In this field of research, replicas are often assumed to be less valued than authentic objects. However, our findings show that this is not the main perspective among participating museum educators. This means that discussions about and support for the use of authentic objects and replicas should take a more fine-grained approach, taking into account the variety of types of objects, stories, and learning goals.

Our study has shown that authenticity has many aspects, and different educators consider different aspects to be important. Educators often agreed on the broad definition of authenticity but laid emphasis on different aspects of this definition. This variation in definition can make it difficult to discuss its importance and application during educational activities. In addition, participants’ ideas about the definition seemed to develop during the interviews, which emphasizes the importance of discussing the topic of authenticity. Finally, it is important to clarify the used interpretation of authenticity within the museum (research) field. One could even argue that the discussion should not be focused on whether an object is authentic or not, but on which factors would make an object more or less authentic, and on the ways we can convey the history and story of an object.

In conclusion, this study shows that museum educators’ perspectives on authenticity and handling objects are largely in line with the existing findings on the importance of using objects to enhance the learning experience during an educational activity. According to science museum educators, using objects can prompt curiosity which improves the learning experience. In addition, being able to touch the objects enables hands-on learning which increases engagement with the object and is therefore beneficial for the learning experience. However, our findings also indicate that museum educators may use a variety of definitions for authenticity. Hence, the choice for the use of authentic objects and replicas and for allowing visitors to touch them is not as clear-cut as might have been suggested by previous studies and will depend largely on context and learning goals.

## Supplemental Material

sj-pdf-1-pus-10.1177_09636625231202617 – Supplemental material for Science museum educators’ views on object-based learning: The perceived importance of authenticity and touch Supplemental material, sj-pdf-1-pus-10.1177_09636625231202617 for Science museum educators’ views on object-based learning: The perceived importance of authenticity and touch by Tirsa de Kluis, Sanne Romp and Anne M. Land-Zandstra in Public Understanding of Science
