# Supplementary material for: Science museum educators’ views on object-based learning: The perceived importance of authenticity and touch
Source: Public Underst Sci. 2023 Nov 2;33(3):325–42. doi: 10.1177/09636625231202617 (PMC10958754; doi:10.1177/09636625231202617)
Supplement: sj-pdf-1-pus-10.1177_09636625231202617 – Supplemental material for Science museum educators’ views on object-based learning: The perceived importance of authenticity and touch [file sj-pdf-1-pus-10.1177_09636625231202617.pdf]

Supplemental material

for

Science Museum Educators' views on Object-Based Learning: The Perceived Importance of Authenticity and Touch

Tirsa de Kluis, Sanne Romp, Anne M. Land-Zandstra*

*Corresponding author: a.m.land@biology.leidenuniv.nl

Public Understanding of Science

S1 – Survey p. 2

S2 – Interview Scheme p. 6

S3 – Codebook Survey p. 8

S4 – Codebook Interview p. 11

S1: Survey

Open spaces for answering have been removed.

The Use of Objects

Do you develop or are you involved in the development of science museum lessons (*educational lessons focused on a specific topic in a museum setting, in line with a school curriculum or methods)?* Yes/No

Do you currently teach science museum lessons, or did you teach science museum lessons in the past? Yes/No

Do you supervise or have you supervised activities (giving a tour, helping children designing or crafting something, demonstrations, interactive talks, shows) in a science museum? *(If answer is No on first 3 questions, go to the end of the survey)* Yes/No

How do you use objects (both authentic and replicas) during your museum lesson/activity? *(Multiple answer options. If answer is "I do not make use of objects", than show only the following question, and go to end of the survey)*

- I do not make use of objects
- I show visitors objects
- I allow visitors to touch and hold objects under my supervision
- Visitors are allowed to look at objects independently
- Visitors are allowed to touch and hold objects independently
- Other: …………………………………………………………………………………

Do you think it is / is not important to use objects during a museum lesson/activity? *(One answer option)*

- It is important to use objects, because ....
- It is not important to use objects, because...

Can you give one or more typical examples of how you use objects in your museum lesson/activity?

What do you use predominantly: authentic objects or replicas? Keep 1 lesson/activity in mind that is most typical for your organization *(One answer option)*

- Authentic objects
- Replicas that are as realistic as possible (e.g. exact casts/copies)
- Less realistic replicas (e.g. 3D prints)
- Both authentic objects and replicas

What is the average distribution of authentic objects and replicas during your museum lessons/activity? *(Answer this question if the answer above is "both authentic and replica". One answer option)*

- ○ Mainly authentic objects
- ○ About as many authentic objects as replicas
- ○ Mainly replicas

Describe details of the distribution of objects during your museum lesson/activity *(not required):*

Touch
Below, a number of statements are shown. Choose 1 answer each time.

Totally disagree	Disagree	Neutral	Agree	Totally agree
O	O	O	O	O

Visitors should be able to touch objects during a museum lesson/activity.

Visitors learn more from a museum lesson/activity when they are allowed to touch objects compared to when they are only allowed to look at objects.

For the learning process, it does not matter whether visitors could touch objects or not.

Visitors are more attracted/aroused/amazed by a museum lesson/activity in which they are allowed to touch objects than a lesson in which they can only look at objects.

It is important that the objects visitors are allowed to touch are authentic.

Explanation (not required):

Authenticity
Below, a number of statements are shown. Choose 1 answer each time.
Caution: the replicas mentioned in the following statements refer to **highly realistic replicas**, such as exact casts.

Totally disagree	Disagree	Neutral	Agree	Totally agree
O	O	O	O	O

Visitors learn more from a museum lesson/activity in which authentic objects are used, compared to replicas.

For the learning process, it does not matter whether the used objects are authentic or replicas.

Visitors become more enthusiastic about a museum lesson/activity that uses authentic objects than those using replicas.

It is important that visitors look at authentic objects during the museum lesson/activity, even if they cannot touch them.

I would rather have visitors touch a replica (substituted object) than have them only look at an authentic object that they are not allowed to touch.

Explanation (not required):

Suppose your museum has made a budget available. There are two options:

1. You may purchase a genuine Tyrannosaurus tooth to display in a showcase during your museum lesson/activity.
2. You may have an exact replica made of this tooth that visitors could touch and pass on.

What would you choose and why?

Imagine that you want to use a vulnerable prehistoric pot during your museum lesson/activity. **What would you do in terms of using the authentic pot versus a replica, and what would you allow visitors to do with the object?**

What do you think is the potential added value of using touchable authentic objects in museum lessons/activities?

- ○ Added value 1 ……………………………………………………
- ○ Added value 2 ……………………………………………………
- ○ Added value 3 ……………………………………………………
- ○ There is no added value

What possible barriers do you experience when using touchable authentic objects in a museum lesson/activity?

- ○ Barrier 1 ……………………………………………………
- ○ Barrier 2 ……………………………………………………
- ○ Barrier 3 ……………………………………………………
- ○ There is no barrier

What do you think is the potential added value of using as realistic as possible, touchable replicas in museum lessons/activities?

- ○ Added value 1 ……………………………………………………
- ○ Added value 2 ……………………………………………………
- ○ Added value 3 ……………………………………………………
- ○ There is no added value

What possible barriers do you experience when using as realistic as possible, touchable replicas in museum lessons/activities?

- ○ Barrier 1 ……………………………………………………
- ○ Barrier 2 ……………………………………………………
- ○ Barrier 3 ……………………………………………………
- ○ There is no barrier

General Information

Are you currently active as a museum educator/developer of museum lessons/activities?
Yes/No

What age group(s) are you most involved with, regarding museum lessons/activities?
(Multiple answer options)

- 4 – 7
- 8 – 10
- 11 – 13
- 14 – 16
- > 16

In which subject area do you teach/develop lessons/activities?

Approximately, how many years of experience do you have in giving/developing museum lessons/activities?

What topics are highlighted in the museum where you are currently employed?

- Physics / Chemistry / Mathematics / Science
- Ethnology
- Biology
- Geography
- Other, namely

In which country is the museum located where you are currently employed?

What is your gender?

- Male
- Female
- Other

Would you be willing to anticipate a follow-up interview by phone? Your email address will be stored separately from your survey answered and will be treated confidentially. It will only be seen and used by the researchers who are directly involved in this study.

- Yes, (email address)
- No

S2: Interview scheme

General questions

1. What is your role within the museum?
2. How many years of experience do you have working as a museum educator?
3. What is your background experience?
4. About which area do you deliver most museum lessons/activities?
5. What ages do you mostly interact with?

Theme 1: Definition of authentic objects

As I mentioned in the introduction, we are interested in the use of objects in museums and possible differences in the use of authentic objects and replicas. Because authentic is not a term that has a clear definition, I would like to first briefly explain what we mean with it within this research. Authentic objects can be real objects, for example objects that originated in nature like fossils. Objects can also be authentic because they are unique, or have a long history. Also, some people see objects as authentic when they have belonged to a famous person.

6. What do you think of this definition?
7. Would you consider an educative model, specially designed for a museum to, for example, illustrate an abstract concept, authentic? Why (not)?
8. Would you consider a taxidermied animal authentic? Why (not)?
9. What is your favorite object you use during museum lessons/activities? Why?
10. Do you see that object as authentic? Why (not)?

Theme 2: Importance of authentic objects and touch

11. Could you describe the typical set-up of a museum lesson or activity you deliver?
12. What do you think is the most educational museum lesson or activity in your museum?
13. Do you use objects during this lesson/activity?
    - If so, could you describe them?
    - How do you use these objects?
    - Would you describe these objects either as replicas or as authentic?
14. Could you give a typical example of how you use objects in the museum lesson/activity?
15. According to you, is it important to use objects during museum lessons/activities? Why (not)?
16. Do you find it important if these objects are authentic?
17. What is the distribution of authentic objects and replica's within this museum lesson/activity?
18. What is the importance of touching objects during museum lessons/activities?
    - Do you think touching objects influences the learning experience?
    - Is this different between visitors of different ages?
    - Does this differ between authentic objects and replicas?

Theme 3: Application of authentic objects

19. Do you have the freedom to choose which objects you want to use in the museum lesson/activity? If so/If you would, what do you base this decision on?
20. Could you describe a situation in which you would use an authentic object in the museum lesson/activity?

21. Could you describe a situation in which you would use a replica in the museum lesson/activity?
22. Do you experience barriers when using objects during the museum lesson/activity? If so, how do you experience this?
23. How would you manage it if the object you want to use in the museum lesson/activity is fragile?
24. If you had to choose between showing an authentic object or giving the opportunity to touch an exact replica, what would you do?

S3: Codebook Survey

Category (#)	Description	Code	*Included terms*/Sub code	Example
**Goal (Q5)**	The goal of using objects during lessons or activities	Engagement	*Interest/Curiosity/Discovery*	It can prompt interest and curiosity (E010, Q5)
			Grab attention	It makes it more easily to explain things, people are more aware and attentive (E006, Q5)
			Amaze/Empathize	And of course you can amaze them, enthuse (N002, Q5)
			Start conversation	By using an object you get a conversation with the visitors (E003, Q5)
			Emotion	Objects speak to emotion, empathy, creativity, etc. (N005, Q5)
		Learning	*Inform/Elaborate/Understand*	Sometimes it's hard for the visitors to really grasp how something looks like or functions. The objects help in understanding and also help visitors to make their own realizations (E003, Q5)
			Senses/Visualization	Because the multi-sensory experience is the deepest and widest (N016, Q5)
		Museum's right to exist	*Original objects/Expectation*	Because we are a Museum! No objects, no museum! (E016, Q5)
		(Museum) Experience	*Meaningful/Memorable*	'Real' objects make experience more meaningful for visitors (E004, Q5)
			History/Reality	You come into direct contact with a certain time frame (N023, Q5)
**Use (Q6+9+15)**	What kind of objects and in what way educators use objects during lessons or activities	Authentic objects	Show (*in showcase*)	If this is an object from the official museum collection I would only let people look at it, as close as possible and then in a small display case (N004, Q15)
			Touch (*supervised*)	With an authentic pot, I might still allow people to touch the side of the pot, but not lift or hold it (E015, Q15)
			Hold/Pass around	During a program about mammoths, I have a piece of mammoth tusk held (N015, Q6)
			No specification	For natural history lessons, animal materials can be used without any problems (N021, Q9)
		Replicas	Show	A cast of an Iguanodon tooth to show the form of the teeth of certain plant eating dinosaurs (E013, Q6)
			Touch	Have replicas available for more in depth study in which the learner can manipulate the object themselves (E009, Q15)
			Take home/Design	The first thing that comes to my mind is that I would like to let visitors make a pot themselves instead of having them look at a pot (N015, Q15)
			No specification	The artifacts are mostly functional and they serve a purpose in the dramatic narrative of the activity (E017, Q9)
		Educational collection	Touch	We have educational collection of specimens that pupils are allowed to manipulate during (various themes of) workshops (E016, Q6)
		Similar object to compare with		It can be compared to contemporary similar objects (these could touch the visitors) (N010, Q15)
		No specification authentic/replica	Touch	For example, I use the objects to make people feel and experience how something feels, how heavy it is, what texture it has, etc. (N008, Q6)

			Investigate	To get students to describe features and make observations (E005, Q6)
			Explain/Illustrate	Also if we have some exhibit, we sometimes use object to explain the phenomena of certain exhibit (E021, Q6)
			Handle equally	I would use the same principles for both objects (E005, Q15)
**Consideration touch or show (Q14)**	If the educator would rather allow visitors to touch a replica or to only look at an authentic object	Show authentic	Impact (*History/Attention*)	The idea that such a tooth was in a real mouth and that it allowed millions of years ago a dinosaur to eat leads to a nice conversation (N007, Q14)
			(Museum)Value	It must always be the goal to present original authentic objects in museums (E002, Q14)
			Loose replica	The exact replica would disappear if it is possible to pass it on (E013, Q14)
			Sustainable	I would choose number 1 because I am concerned about the durability of a commonly used replica (N008, Q14)
			With restriction (*touch replica*)	Why not both? The original object is important because it is used for research …If would be perfect if there could also be a replica to touch (E007, Q14)
			No explanation	I think I prefer the real object in the display case over the replica. But the replica in a display case; I don't like that. (E016, Q14)
		Touch replica	Learning *(Investigate yourself /Senses/Details/Remember*)	The act of touching and manipulating is more powerful as a learning and memory making tool (E011, Q14)
			More accessible (*Cheaper/Blind*)	It's also better for people who have challenges with their sight (E003, Q14)
			Engagement (*Amaze*)	With small children I would rather go for the replica and hold, because then it is very important to wonder. (N001, Q14)
			Tell it is authentic	Would I be tempted to purchase the replica BUT tell people it was authentic? (E008, Q14)
			With restriction (*show authentic*)	If I had to choose, I would go for the replica. Assuming that a similar original object can be seen in the collection or exhibition (N017, Q14).
			No explanation	In this particular case, I find this more important (N003, Q14)
**Touchable authentic objects (Q16+17)**	The added value and barrier of touchable authentic objects	Object characteristics	*+ Unique object*	The feeling you get in touch with something special or unique (E002, Q16)
			+ History/ Authenticity	Getting in touch with history (E001, Q16)
			+ Material information (Details)	The sense of touch provides information about material etc. (N003, Q16)
			- Vulnerable (Supervision/Safety)	Vulnerability; you have to pay more attention and children can use it a little less freely than with replicas (N002, Q17)
		Impact visitor	*+ Learning (Senses)*	Enhancing the learning experience by involving several senses (E001, Q16).
			+ Connection (to the past)	Touching original give a special connection to the past (E012, Q16)
			+ Long term effects	The experience will be remembered for longer (E023, Q16).
			+ Experience (Engagement, Meaningful, Emotion)	Other experience than in a normal lesson (N019, Q16)
			- Misunderstood value/Careless	People cannot always tell what the object is at thus begins to play around with the object and making their own explanation to what it is (E010, Q17)
			- Less clear view	Authentic objects sometimes give a less clear picture of what you want to show than replicas (N002, Q17)

		Museum characteristics	+ *Appreciate collection*<br><br><br>- *Accessibility (Expensive, Irreplaceable)*<br>- *Conservation*<br>- *Rules/Ethics/Stealing*	Touching originals is something which has to be in the museum, it cannot be done digitally (E012, Q16).<br>Limited number of objects - hard for all to access everything (E005, Q17)<br>Preservation conditions (E019, Q17)<br>Ethics (mishandling objects with symbolic importance for some communities) (E001, Q17)
		Other	+<br>-	Power (E011, Q16)<br>More constraints (E004, Q17)
**Touchable replicas (Q18+19)**	The added value and barrier of touchable replicas	Object characteristics	+ *Less vulnerable (allowed to touch)*<br>+ *Multiple replicas*<br><br>+ *Material information (Details)*<br><br>+ *Sustainable*<br>+ *Authentic*<br>- *Lack of authenticity/ Accuracy (misleading)*<br><br>- *Vulnerable (supervision)*<br><br>- *Safety*	Stronger and withhold more wear and tear (E014, Q18).<br>Several replicas at the same time available for many visitors (E019, Q18)<br>With replicas you can observe details of the object better (E021, Q18).<br>Sustainable (N005, Q18)<br>Real and authentic (N009, Q18)<br>Bad reproduction or reproduction with a wrong material (misinterpretation) (E019, Q19)<br>Additional deployment of personnel if necessary (N008, Q19)<br>H&S implications (E009, Q19)
		Impact visitor	+ *Learning (Senses)*<br><br>+ *Long term effect*<br><br>+ *Experience (Engagement, Meaningful, Emotion)*<br><br>- *Loose interest/Respect*<br>- *Disappointment*	More intensive learning experience through the use of multiple senses (N002, Q18)<br>Visitors remember their experiences better (N008, Q18)<br>The experience remains, especially next to originals next to it in the display case (N003, Q18)<br>Visitors are less careful with it (N010, Q19)<br>It is not real, which might be a bit of a disappointment to the visitor. Therefore, they might learn/remember less of it (E023, Q19)
		Museum characteristics	+ *Allowed/Cheaper*<br>- *Accessibility/Budget*<br><br>- *Stealing*	Price – cheaper than original (E004, Q18)<br>Replicas are usually expensive to purchase (N024, Q19)<br>Supervise to prevent theft (N004, Q19)
		Other	+<br><br><br>-	In the case of target group children: it can become a game where it is supposedly real (but always state that it is not) (N004, Q18)<br>Practical (N009, Q19)

S4: Codebook interview

Multiple codes can be assigned to one quotation.

**Category**	**Code**	**Description**	**Sub-code 1**	**Sub-code 2 (Optional)**	**Sub-code 3 (Optional, engagement)**	**Description**	**Examples**
Institute	Topics	Topics of the museum lessons and activities					“So it has been everything from evolution, of course with a lot of that happening at the zoological museum, but also whatever topics they could be interested in having.” (quotation I1)
	Target group	Target group on which the museum lessons and activities are aimed					“Children between eight and fourteen bring their parents, or their grandparents, and we are specifically there for them as well. So we are there for students and families, we are not a children-museum.” (quotation I9)
	Professional experience	Experience of the museum educator	Teaching			Educator discusses experience with teaching in schools	“I have also worked in secondary education. I stood in front of a classroom. I gave history and social studies.” (quotation I9)
			Education	Scientific<br>Science communication Museum related		Educator discusses own education, either a specific research area, a science communication related study, a museum related study, or a combination thereof	“I studied to be a geography teacher and afterwards I studied social-geography and geo-communication, which is a kind of science communication.” (quotation I3)
	Role	Role the museum educators (or the institute in general) takes on during the museum lesson or activity	Providing information			Educators’ main role is providing information to the participants	“We always had a complete story and then we grabbed objects ourselves, like ‘this long ago Texel emerged from the Ice Age, mammoths walked around here, look this is from a mammoth’.” (quotation I10)
			Providing encouragement/interaction			Educators’ main role is providing encouragement or interaction with the visitor	“So it is not a monologue, it always has to be a dialogue between the participants and the guide.” (quotation I12)
			Providing context	Story<br>Set-up		Educators’ main role is providing context, either in the form of how the lesson or activity is set-up or in the story he/she tells during the activity	*Story:* “So this idea about the narrative is really important.” (quotation I11)<br><br>*Set-up:* “It is only small courses, they do not stay for a lot of hours. So the small children, the school classes, they would only have like forty-five minutes and the older ones would have one and a half hour [..]” (quotation I1)

	Object choice	Process of choosing objects to use in the museum lesson or activity					"Well we will start by looking at what we need in order to teach the kids what we want to teach them. So if we are going to teach them the one where they count the teeth, then of course we will have some of the different animals with the different amounts of teeth in their mouth." (quotation I1)
	Barriers	Barriers for the use of objects that stem from the institute	Collection			There are rules for the collection that hinder educators in their object choice	
			Costs			There is a certain budget that the educator needs to honor	
			Practicality			Educator cannot use certain objects for practical reasons	
			Ethics			Institute deems it unethical to use certain objects	
			Rarity			Educator is not allowed to use an object because of its rarity	

Object	Authenticity	Application and definition of authenticity	Definition	Real/From nature<br>Unique<br>Rarity<br>Long history<br>Belonging to a famous person		How the educator defines the term authenticity	Real/From nature: "I think normally we would only call it authentic if it is really something from nature and not something man-made." (quotation I1)<br><br>Unique: "But we do work with authentic objects in the sense of, some of them were created for our ateliers and they are unique in their kind." (quotation I4)<br><br>Rarity: "What makes it authentic for me is that it is special, you cannot find it at a supermarket or something like that." (quotation I4)<br><br>Long history: "If it has a long history and many things have happened to it, then there are only a few objects that lived through all those events, to say it like that. So I get that it then becomes more authentic." (quotation I2)<br><br>Belonging to a famous person: "A famous person is something that we do not have here actually, because we have all these archaeological objects and we do not know to whom they belonged of course." (quotation I8)
			Application of authentic objects	Added value<br>Educational value<br>Appearance<br>Reason for existence		Reasons for choosing an authentic object (or authentic context) over a replica	General example (place this under sub-code 1 and if there is extra information a sub-code 2 can be added): "Well we would always prefer the authentic." (quotation I1)
			Application of replicas	Added value<br>Educational values<br>Appearance		Reasons for choosing a replica over an authentic object	General example (place this under sub-code 1 and if there is extra information a sub-code 2 can be added): "We only, you know, have the copies whenever we need them, but sometimes it is easier to have a copy." (quotation I1)

			Distinction	Importance (Yes/No)<br>Similar object<br>Use equally		Way in which educators distinguish authentic objects and replicas for their audience	Importance (Yes/No): “You definitely, they are all interested in knowing the difference. And also if they are just walking around the museum and looking at all of the animals and the skeletons, then they would also be really interested in what is real and 'oh does it look like that when you get up close', and you know.” (quotation I1)<br><br>Similar object: “But if you can do both, it would always be the best.” (quotation I1)<br><br>Use equally: “Even with those plastic objects, that state ‘made in China’, even of those objects they will ask, and they will do that to the age of ten, ‘is it real?’ Well then I ask ‘what do you think?’ ’Well no, I don’t think so’, they will say, ‘because it says ‘made in China’’. But still, if you see how they treat them afterwards, that is just as careful as the fossils lying next to it.” (quotation I3)
	Use	What kind of objects and in what way educators use objects during lessons or activities	Authentic	Impact touch	Amaze<br>Respect<br>Interest	Impact of touching an authentic object on engagement	Amaze: “I think it is just 'wow, I touched something that was made 2000 years ago by someone'. I think that that makes a difference.” (quotation I8)<br><br>Respect: “It is amazing that things do not break. So most of the replicas have been in use for ten years now in these suitcases, and it is still the first one we bought. So they are very very careful with it.” (quotation I8)<br><br>Interest: “Yeah, and we actually have a replica from a T. rex tooth which is not authentic but we still like to show it around because it is very impressive and it is interesting for people to know something about the T. rex and you can explain certain things using this tooth.” (quotation I12)
				Impact show	Amaze<br>Respect<br>Interest	Impact of showing an authentic object on engagement	
				Impact handle	Amaze<br>Respect<br>Interest	Impact of handling an authentic object on engagement	
				Impact no specification	Amaze<br>Respect<br>Interest	Impact of using an authentic object on engagement (no further specification on touch/show/handle)	
			Replica	Impact touch	Amaze<br>Respect<br>Interest	Impact of touching a replica on engagement	
				Impact show	Amaze<br>Respect<br>Interest	Impact of showing a replica on engagement	

				Impact handle	Amaze<br>Respect<br>Interest	Impact of handling a replica on engagement	
				Impact no specification	Amaze<br>Respect<br>Interest	Impact of using a replica on engagement (no further specification on touch/show/handle)	
			No specification	Impact touch	Amaze<br>Respect<br>Interest	Impact of touching an object (no specification on authenticity) on engagement	
				Impact show	Amaze<br>Respect<br>Interest	Impact of showing an object (no specification on authenticity) on engagement	
				Impact handle	Amaze<br>Respect<br>Interest	Impact of handling an object (no specification on authenticity) on engagement	
				Impact no specification	Amaze<br>Respect<br>Interest	Impact of using an object (no specification on authenticity) on engagement (no further specification on touch/show/handle)	
	Goal	The goal of using objects during lessons or activities	Authentic	Impact touch	Connect to ‘own world’<br>Catch attention<br>Take students seriously<br>History<br>Scientific literacy	Goal intended by educator for touching an authentic object with regards to engagement	Connect to ‘own world’: “You can connect it very good to normal life because the dinosaur only has teeth in the front and not in the back. So you can ask how did it chew and what would happen if you had the same teeth and eat a carrot, or something like that. So you can connect it with normal life experience of the visitors.” (quotation I12)<br><br>Catch attention: “But I am perhaps a bit more oldschool in that I like real objects as a way to then lead into doing something. But I like the idea about you give people something and they go 'oh that is quite interesting'.” (quotation I11)<br><br>Take students seriously: “Yeah, but on the other hand, if a student insists on using gloves then we are
				Impact show	Connect to ‘own world’<br>Catch attention<br>Take students seriously<br>History<br>Scientific literacy	Goal intended by educator for showing an authentic object with regards to engagement	
				Impact handle	Connect to ‘own world’<br>Catch attention<br>Take students seriously<br>History<br>Scientific literacy	Goal intended by educator for handling an authentic object with regards to engagement	

				Impact no specification	Connect to ‘own world’<br>Catch attention<br>Take students seriously<br>History<br>Scientific literacy	Goal intended by educator for using an authentic object with regards to engagement (no further specification on touch/show/handle)	not going to try and push them in that direction. We are going to say that is completely cool.” (quotation I7)<br><br>History: “You can observe that historical sensation in people if they can really touch it.” (quotation I9)<br><br>Scientific literacy: “And then they learn, that is the scientific literacy, that there are just many things that we do not know. Something can be said, using arguments, like it belongs to that species or there are also arguments that say it is a separate species. The main thing is that we do not know everything, but based on arguments and discussing with each other and investigating, you can possibly find the correct answer.” (quotation I2)
			Replica	Impact touch	Connect to ‘own world’<br>Catch attention<br>Take students seriously<br>History<br>Scientific literacy	Goal intended by educator for touching a replica on engagement	
				Impact show	Connect to ‘own world’<br>Catch attention<br>Take students seriously<br>History<br>Scientific literacy	Goal intended by educator for showing a replica with regards to engagement	
				Impact handle	Connect to ‘own world’<br>Catch attention<br>Take students seriously<br>History<br>Scientific literacy	Goal intended by educator for handling an authentic object with regards to engagement	
				Impact no specification	Connect to ‘own world’<br>Catch attention<br>Take students seriously<br>History<br>Scientific literacy	Goal intended by educator for using an authentic object with regards to engagement (no further specification on touch/show/handle)	
			No specification	Impact touch	Connect to ‘own world’<br>Catch attention<br>Take students seriously<br>History<br>Scientific literacy	Goal intended by educator for touching an object (no specification on authenticity) with regards to engagement	
				Impact show	Connect to ‘own world’<br>Catch attention<br>Take students seriously<br>History<br>Scientific literacy	Goal intended by educator for showing an object (no specification on authenticity) with regards to engagement	
				Impact handle	Connect to ‘own world’<br>Catch attention<br>Take students seriously<br>History<br>Scientific literacy	Goal intended by educator for handling an object (no specification on authenticity) with regards to engagement	

				Impact no specification	Connect to ‘own world’<br>Catch attention<br>Take students seriously<br>History<br>Scientific literacy	Goal intended by educator for using an object (no specification on authenticity) with regards to engagement (no further specification on touch/show/handle)	
	Barriers	Barriers for the use of objects that stem from the object itself	Fragility			Object is too fragile to use	
			Safety			Object is not safe to use	
			Rarity			Object is too rare to use	
			Sustainability			Object is not sustainable	

Visitor	Learning	Way in which learning occurs because of the use of objects (only use when learning or a synonym for learning is explicitly mentioned)	Touch	Inquiry			Inquiry: “That is a lesson which uses inquiry-based learning, so they will investigate their own stone.” (quotation 8:77)<br><br>Multiple senses: “Especially when you have a replica that can be held. Then you use even more senses, that works much better than having a real T. rex tooth in a display.” (quotation 8:41)<br><br>Added information: “Sometimes it is much more grainy. That also says something about the kind of stone that it is.” (quotation 8:48)<br><br>Adding to curriculum: “So whatever is tough for the teachers, that is what we normally try to do in our lessons. So they could come to the museum and have some of the hard stuff. So they did not have to at home at the school.” (quotation 5:48)<br><br>Learning styles: “I think that for additional needs or any kinds of neurodiverse audiences it provides a tactile experience, particularly if there is any sort of visual impairment. It can really generate a learning experience where perhaps there would have been a barrier.” (quotation 11:13)<br><br>Memorisable: “I think it makes people remember it mostly [..]” (quotation 13:39)
				Multiple senses			
				Added information			
				Adding to curriculum			
				Learning styles			
				Memorisable			
			Show	Added information			
				Adding to curriculum			
				Learning styles			
				Memorisable			
			Handle	Inquiry			
				Multiple senses			
				Added information			
				Adding to curriculum			
				Learning styles			
				Memorisable			
			No specification	Inquiry			
				Multiple senses			
				Added information			
				Adding to curriculum			
				Learning styles			
				Memorisable			
	Barriers	Barriers for the use of objects that stem from the visitor	Safety				
			Personal reasons				
			Ethics				
